# Speech Acoustic Features: A Comparison of Gay Men, Heterosexual Men, and Heterosexual Women

**DOI:** 10.1007/s10508-020-01665-3

**Published:** 2020-03-31

**Authors:** Alexandre Suire, Arnaud Tognetti, Valérie Durand, Michel Raymond, Melissa Barkat-Defradas

**Affiliations:** 1 CNRS, IRD, EPHE, Institut des Sciences de l’Evolution, University of Montpellier, Montpellier, France; 2 Institute for Advanced Study in Toulouse, 21 Allée de Brienne, 31015 Toulouse, France; 3 Department of Clinical Neuroscience, Karolinska Institutet, Stockholm, Sweden

**Keywords:** Speech, Voice, Acoustics, Sexual orientation, Testosterone levels, Gender atypicality

## Abstract

Potential differences between homosexual and heterosexual men have been studied on a diverse set of social and biological traits. Regarding acoustic features of speech, researchers have hypothesized a feminization of such characteristics in homosexual men, but previous investigations have so far produced mixed results. Moreover, most studies have been conducted with English-speaking populations, which calls for further cross-linguistic examinations. Lastly, no studies investigated so far the potential role of testosterone in the association between sexual orientation and speech acoustic features. To fill these gaps, we explored potential differences in acoustic features of speech between homosexual and heterosexual native French men and investigated whether the former showed a trend toward feminization by comparing theirs to that of heterosexual native French women. Lastly, we examined whether testosterone levels mediated the association between speech acoustic features and sexual orientation. We studied four sexually dimorphic acoustic features relevant for the qualification of feminine versus masculine voices: the fundamental frequency, its modulation, and two understudied acoustic features of speech, the harmonics-to-noise ratio (a proxy of vocal breathiness) and the jitter (a proxy of vocal roughness). Results showed that homosexual men displayed significantly higher pitch modulation patterns and less breathy voices compared to heterosexual men, with values shifted toward those of heterosexual women. Lastly, testosterone levels did not influence any of the investigated acoustic features. Combined with the literature conducted in other languages, our findings bring new support for the feminization hypothesis and suggest that the feminization of some acoustic features could be shared across languages.

## Introduction

The gender atypicality hypothesis suggests that gender atypical traits in homosexuals could be used as cues to indicate sexual orientation. Differences between heterosexual and homosexual individuals have thus been studied on a diverse set of traits such as face (e.g., Freeman, Johnson, Ambady, & Rule, 2010; González-Álvarez, 2017; Lyons, Lynch, Brewer, & Bruno, 2014; Rieger, Linsenmeier, Gygax, Garcia, & Bailey, 2010; Skorska, Geniole, Vrysen, McCormick, & Bogaert, 2015; Wang & Kosinski, 2018), olfaction (e.g., Sergeant, Dickins, Davies, & Griffiths, 2007), behavior (e.g., Ambady, Hallahan, & Conner, 1999; Rieger, Linsenmeier, Gygax, & Bailey, 2008; Valentova, Rieger, Havlicek, Linsenmeier, & Bailey, 2011), cognition (e.g., Neave, Menaged, & Weightman, 1999; Xu, Norton, & Rahman, 2017), and voice (e.g., Gaudio, 1994; Munson, McDonald, DeBoe, & White, 2006b; Pierrehumbert, Bent, Munson, Bradlow, & Bailey, 2004; Rendall, Vasey, & McKenzie, 2008). In addition to the fact that homosexuals exhibit traits that differ from those of heterosexuals, it has been shown that some of them, such as specific neural processes (LeVay, 1991; Savic, Berglund, & Lindstrom, 2005) or specific childhood behaviors (Alanko et al., 2010; Bailey & Zucker, 1995), displayed values shifted toward those of the opposite sex, i.e., a feminization in homosexual men and a masculinization in homosexual women (Pierrehumbert et al., 2004). Moreover, studies have shown that both men and women are able to accurately assess sexual orientation from both sexes from various features such as the face or body movements (Ambady et al., 1999; Rieger et al., 2010; Valentova et al., 2011; Wang & Kosinski, 2018). These findings emphasize the idea that specific phenotypic traits may be influenced by sexual orientation and may be used as cues to detect or advertise it.

Another important trait that seems to be influenced by sexual orientation and used as a cue to assess is speech. (For a detailed review, see Munson & Babel, 2007.) For example, popular stereotypes regarding the speech of homosexual men generally attribute speech patterns characteristic of the opposite sex, i.e., a broadly feminized speech, such as a higher fundamental frequency (i.e., F0, the acoustic correlate of voice pitch) and a greater variation in the intonation (i.e., F0-SD, the local variations of F0 throughout speech, henceforth, referred to as vocal modulation) (Cartei & Reby, 2012; Munson & Babel, 2007). Although there is no clear evidence that the mean fundamental frequency differs between homosexual and heterosexual men (Gaudio, 1994; Lerman & Damsté, 1969; Munson et al., 2006b; Rendall et al., 2008; Rogers, Jacobs, & Smyth, 2001; Smyth, Jacobs, & Rogers, 2003; but see Baeck, Corthals, & Borsel, 2011), results toward differences in pitch modulation patterns are more controversial: Some studies have found that homosexual men displayed greater variations in intonation, with values shifted toward those of women (Baeck et al., 2011; Gaudio, 1994), while others did not find any difference (Levon, 2006; Rogers et al., 2001). Spectral measures of fricatives also seemed to be influenced by sexual orientation (Munson, Jefferson, & McDonald, 2006). For instance, homosexual men produce higher peak frequency and longer duration values for /s/ (Linville, 1998) and these speech characteristics are associated with “gayer-sounding” voices by listeners (Mack & Munson, 2012). Lastly, homosexual men seem to produce a more expanded vowel space than heterosexual men for some specific vowels (Rendall et al., 2008), hyper-articulation being commonly found in female speech (Pierrehumbert et al., 2004).

Aside these acoustic speech features, other characteristics could vary with sexual orientation, such as vocal breathiness and roughness that are, respectively, captured by the harmonics-to-noise ratio (HNR) and the jitter. Indeed, both components are sexually dimorphic as women exhibit significantly higher values of HNR (i.e., lower “breathy” voices) and lower values of jitter (i.e., lower “rougher” voices) than men (Graddol & Swann, 1989; Van Borsel, Janssens, & De Bodt, 2009). Although vocal breathiness has been suggested to be an important component of femininity for female voices (Van Borsel et al., 2009), significant relationships in vocal attractiveness for both sexes have been reported (e.g., Xu, Lee, Wu, Liu, & Birkholz, 2013), while vocal roughness has been found to be positively associated with male vocal attractiveness (Hughes, Dispenza, & Gallup, 2004). Such results suggest that vocal breathiness and roughness may play a role in the qualification of masculine versus feminine sounding voices, thus questioning homosexuals’ vocal breathiness and roughness within this continuum. In line with the speech feminization hypothesis, homosexual men could indeed potentially exhibit higher values of HNR and lower values of jitter, but, so far, no studies have tackled this issue.

Researches have tried to assess if the feminized traits in homosexual men can be attributable to proximate mechanisms such as the differences in sex hormone levels. Testosterone, a male sex hormone, has thus been intensively studied as it was found to be associated, for instance, with facial (e.g., Penton-Voak & Chen, 2004; Pound, Penton-Voak, & Surridge, 2009; Roney, Hanson, Durante, & Maestripieri, 2006) and behavioral masculinity (e.g., Apicella et al., 2008; Archer, 2006; Booth, Shelley, Mazur, Tharp, & Kittok, 1989). Concerning acoustic characteristics, several studies have found a negative relationship between fundamental frequency and testosterone levels in men (Dabbs & Mallinger, 1999; Evans, Neave, Wakelin, & Hamilton, 2008; Hodges-Simeon, Gurven, & Gaulin, 2015; Puts, Apicella, & Cardenas, 2012). Although little is known about their physiological mechanisms, both the HNR and jitter have also been suggested to be sensitive to hormonal influx as they both relate to the oscillations of the vocal folds, which possess receptors to circulating androgens (Pisanski et al., 2016). Although evidence of a difference in testosterone levels between homosexual and heterosexual men is inconsistent (Meyer-Bahlburg, 1977, 1984), testosterone may still mediate the relationship between sexual orientation and the aforementioned vocal speech features, which has received little attention so far.

Finally, most of the studies that investigated the link between sexual orientation and speech characteristics have been conducted with native English speakers (e.g., Gaudio, 1994; Linville, 1998; Pierrehumbert et al., 2004; Rendall et al., 2008; see also Baeck et al., 2011; Valentova & Havlíček, 2013 for examples with Dutch and Czech men). This calls for further cross-linguistic examinations as numerous studies have unveiled important differences in vocal quality (i.e., the set of acoustic characteristics linked to a particular voice) across languages (e.g., Andreeva et al., 2014; Keating & Kuo, 2012; Traunmüller & Eriksson, 1995; Zimmerer, Jügler, Andreeva, Möbius, & Trouvain, 2014). Consequently, communities of homosexual men could potentially differ in their specific vocal speech features across different languages.

In this context, the goal of the present study was to provide further details on the potential differences between homosexual and heterosexual men’s speech in an underrepresented population in the literature (i.e., French men). We investigated the effect of sexual orientation on four sexually dimorphic acoustic parameters (F0, F0-SD, jitter, and HNR) and examined whether homosexual men’s vocal characteristics showed a feminization by comparing theirs with that of heterosexual women. Lastly, we examined the potential role of testosterone in the association between speech acoustic features and sexual orientation.

## Method

### Participants

The French National Commission of Informatics and Liberties approved all protocols used in this study (CNIL number 1261003). Participants were recruited by means of flyers handed out as well as advertisements posted on public and private locations in the city of Montpellier, France. In order to recruit as much as possible homosexual males, we contacted the local LGBTQ community to help advertise the study as well as directly advertising it in known local gay bars. All participants gave a written consent prior to the study and were given a financial compensation for their participation. In total, 150 women and 180 men participated in the study. All participants completed a questionnaire assessing their sexual orientation (i.e., they had to state whether they considered themselves as being homosexual, heterosexual, bisexual, or other), their nationality, age, relationship status (single vs. in relationship), socioeconomic status (level of education and monthly income) as well as country of birth of their parents and grandparents.

### Measures and Procedure

#### Speech Samples and Acoustic Analysis Procedure

##### Recordings

Recordings took place in a quiet room in our laboratory at the University of Montpellier. All recordings took place between 2:00 p.m. and 5:00 p.m. Each participant heard the French version of the story “The North Wind and the Sun” from the International Phonetic Association and were asked to tell the story back to the research assistant. The rationale for using semi-spontaneous speech is that it is more ecologically valid than sustained vowels or read speech while controlling for semantic content, as the latter produce very different acoustic speech characteristics that do not represent how an individual vocally behaves in social interactions (Laan, 1997; Suire, Raymond, & Barkat-Defradas, 2018). Speech samples were recorded using a linear PCM recorder (DR-O7 MKII, Tascam©) with a sampling rate of 22 kHz, 16-bit, mono, and then saved as.wav files. To control for intensity, participants were asked to speak within a constant distance of 15 cm from the recorder.

##### Speech Analyses

Because origin (e.g., Ordin & Mennen, 2017; Zimmerer et al., 2014) and language (e.g., Andreeva et al., 2014; Keating & Kuo, 2012; Traunmüller & Eriksson, 1995) influence speech characteristics and vocal quality parameters, we only analyzed participants who were native French speakers with European ascendants. We also only focused on participants who declared themselves as homosexual and heterosexual (we excluded those who declared to be bisexual or other). The final sample size resulted in 48 heterosexual men (age *M* ± SD = 26.18 ± 5.41 years), 58 homosexual men (26.38 ± 5.06 years), and 54 heterosexual women (24.85 ± 4.34 years).

In total, we analyzed these 160 speech samples with the Praat© software (Paul Boersma and David Weenink, Phonetic Sciences, University of Amsterdam, www.praat.org). Pitch floors were set to 75 Hz with a ceiling of 300 Hz for both heterosexual and homosexual men and 85–400 Hz for heterosexual women. All other settings were kept as default. For each participant, we extracted four acoustic parameters: mean fundamental frequency (F0, in Hz), its variations (F0-SD, in Hz), the jitter (%), and the HNR (in dB).

The mean fundamental frequency is the perceptual correlate of the vocal pitch, while its variations are the perceptual correlate of micro-intonation patterns. HNR is the perceptual correlate of vocal breathiness, which corresponds to a ratio between periodic components (i.e., the harmonics) and a non-periodic component (i.e., noise) comprising a segment of voiced speech (Teixeira, Oliveira, & Lopes, 2013). More specifically, this ratio reflects the efficiency of speech production. The greater the flow of air expelled from the lungs into energy of vibration of the vocal cords, the greater the HNR, which is perceptually associated with a more sonorant and harmonic voice. On the contrary, a lower HNR is generally associated with a perceptually asthenic, dysphonic, and breathier voice. Vocal roughness can be captured by the jitter, a measure of the F0 disturbance, which is defined as the parameter capturing the frequency variation from cycle to cycle in the sound wave (Rabinov, Kreiman, Gerratt, & Bielamowicz, 1995). More specifically, the jitter measures the control of the vocal folds during successive periods of oscillations. The higher the jitter, the “rougher” sounds the voice.

#### Saliva Collection and Testosterone Assays

Testosterone levels (henceforth T-levels) were measured in saliva samples (pg/ml). This noninvasive technique has been previously validated and yields T-levels that are highly correlated with serum levels (Ellison, 1988). At the beginning of the experiment, one labeled tube and straw (Salicaps kits, IBL-Hamburg) was given to each participant to collect saliva. Participants were asked not to eat, drink (except plain water), smoke, chew gum, or brush their teeth for 1 h before each session so as to avoid saliva contamination. Samples were kept cold during the duration of the experiment and then stored at − 80 °C before being analyzed by Luminescence ImmunoAssay (LIA) technique, using LIA Testosterone kits (IBL, Hamburg). The assay of each sample was replicated twice, and only measures for which inter-assay CV was lower than 10% were used.

### Statistical Analysis

In order to examine the potential influence of T-levels and sexual orientation on men’s speech, we performed four linear models, one for each acoustic parameter studied. Each acoustic parameter was used as a response variable. To investigate the effects of sexual orientation and test the hypothesis of feminization on these vocal features, we used an explanatory variable called “SexOr” that considers both sex and sexual orientation with three modalities: heterosexual men, homosexual men, and heterosexual women. T-level was added as another explanatory variable. To investigate whether T-level mediates the association between acoustic features and sexual orientation, we also added the interaction between T-level and “SexOr.” When the interaction was not statistically significant, we removed it from the linear models. Age, monthly income, level of education, and relationship status were added as confounding variables. All continuous variables (T-level, age, income, and education) were standardized. Then, to assess if homosexual men displayed vocal features with values shifted toward those of heterosexual women, post hoc analyses (Tukey HSD tests) were performed to compare which category (i.e., heterosexual men, homosexual men, and heterosexual women) differ from one another. Thresholds of significance were corrected for the number of models and post hoc comparisons using the Bonferroni method.

In order to assess the overall difference on speech acoustic features between heterosexual and homosexual men and to examine whether homosexual men’s vocal features are shifted toward those of women, we conducted a linear discriminant analysis (LDA). LDA attempts to model whether a set of variables (here F0, F0-SD, Jitter, and HNR) is effective in predicting category membership (here heterosexual men, homosexual men, and heterosexual women). In other words, we used a LDA to find the linear combinations of the four acoustic features that gives the more accurate separation between the three categories of participants. Then, individuals’ coordinates were computed from the two linear discriminant functions. Those coordinates were used to produce a continuous axis of vocal femininity and masculinity to determine where homosexual men were positioned within this axis. The coordinates of the three groups were then entered in linear models followed by post hoc comparisons (Tukey HSD tests) to assess the overall difference in acoustic speech features.

All statistical analyses were performed under the R software (version 3.1.2).

## Results

Descriptive statistics of all acoustic parameters and T-levels are shown in Table 1.

**Table 1:** Descriptive statistics of mean F0, F0-SD, jitter, HNR, speaking time, and T-levels for heterosexual men and women and homosexual men

	Heterosexual men (*n* = 48) *M* ± SD	Homosexual men (*n* = 58) *M* ± SD	Heterosexual women (*n* = 54) *M* ± SD
F0 (Hz)	118.61 ± 16.74	116.52 ± 13.91	205.89 ± 17.57
F0-SD (Hz)	14.11 ± 4.43	18.22 ± 3.88	32.35 ± 6.60
Jitter (%)	2.63 ± 0.67	2.43 ± 0.57	1.81 ± 0.22
HNR (dB)	10.06 ± 1.42	10.86 ± 1.31	13.68 ± 1.12
Speaking time (s)	68.47 ± 30.10	63.49 ± 33.41	67.58 ± 33.66
T-levels (pg/ml)	137.61 ± 61.97	136.65 ± 53.50	33.08 ± 21.25

The interactions between T-level and “SexOr” did not have a significant effect on mean F0 (*F*(2, 150) = 2.31, *p* = .10), F0-SD (*F*(2, 150) = 0.07, *p* = .93), jitter (*F*(2, 150) = 0.24, *p* = .78), and HNR (*F*(2, 150) = 0.22, *p* = .79). These interactions were thus subsequently removed from the linear models. “SexOr” showed a significant effect on mean F0 (*F*(2, 152) = 225.07, *p* < .001, Table 2), F0-SD (*F*(2, 152)= 95.94, *p* < .001, Table 3), jitter (*F*(2, 152) = 13.59, *p* < .001, Table 4), and HNR (*F*(2, 152) = 55.64, *p* < .001, Table 5). Post hoc comparisons showed that all acoustic characteristics of heterosexual women were significantly different from both heterosexual and homosexual men (all *p* < .01). In addition, homosexual men displayed a significantly higher F0-SD and HNR than heterosexual men (respectively *t*(157) = − 4.48, *p* < .001; *t*(157) = − 2.97, *p* < .01), with values shifted toward those of heterosexual women (Tables 3 and 5). Mean F0 and jitter did not differ between homosexual and heterosexual men (respectively *t*(157) = 0.51, *p* = .86; *t*(157) = 1.79, *p* = .17). Age had a significant positive effect on F0-SD and jitter (respectively *F*(1, 152) = 7.82, *p* < .01; *F*(1, 152) = 10.82, *p* < .01, Tables 3 and 4). Other control variables had no influence on any of the acoustic features under study (*p* > .05, Tables 2, 3, 4, and 5).

**Table 2:** Linear model examining the influence of sexual orientation and sex on mean F0

*R*^2^ = 87%	*β*	SE	*F*	*p*
Intercept	116.67	2.76		
SexOr			225.07	< .001
Heterosexual men/homosexual men	1.67	3.25		
Heterosexual women/homosexual men	85.29	4.28		
Age	− 0.18	0.28	0.44	.50
Testosterone	− 0.03	0.03	1.70	.19
Monthly income	0.38	0.63	0.36	.54
Education	− 0.10	0.41	0.06	.80
Relationship status			0.78	.38
Yes/no	2.34	2.65		

For each variable, the estimate (*β*), standard error of the mean (SE), the *F* and the *p* values associated from the Fisher test of the comparison between the full model and the model without the factor are given. For the categorical variables “SexOr” and “Relationship status,” the estimates are given for one category compared to the reference category (SexOr: Homosexual men; Relationship status: No). *R*^2^ is the variance explained by the model. Sample size: *N*_Heterosexual men_ = 48; *N*_Homosexual men_ = 58, *N*_women_ = 54

**Table 3:** Linear model examining the influence of sexual orientation and sex on F0-SD

*R*^2^ = 71.3%	*Β*	SE	*F*	*P*
Intercept	17.71	0.85		
SexOr			95.94	< .001
Heterosexual men/homosexual men	− 4.48	0.99		
Heterosexual women/homosexual men	13.85	1.32		
Age	0.24	0.09	7.82	< .01
Testosterone	< − 0.01	<0.01	0.19	.65
Monthly income	0.16	0.19	0.70	.40
Education	0.08	0.13	0.37	.54
Relationship status			2.31	.13
Yes/no	1.24	0.81		

For each variable, the estimate (*β*), standard error of the mean (SE), the *F* and the *p* values associated from the Fisher test of the comparison between the full model and the model without the factor are given. For the categorical variables “SexOr” and “Relationship status,” the estimates are given for one category compared to the reference category (SexOr: Homosexual men; Relationship status: No). *R*^2^ is the variance explained by the model. Sample size: *N*_Heterosexual men_ = 48; *N*_Homosexual men_ = 58, *N*_women_ = 54

**Table 4:** Linear model examining the influence of sexual orientation and sex on jitter

*R*^2^ = 33.8%	*β*	SE	*F*	*p*
Intercept	2.41	0.09		
SexOr			13.59	< .001
Heterosexual men/homosexual men	0.18	0.10		
Heterosexual women/homosexual men	− 0.52	0.13		
Age	0.03	< 0.01	10.82	< .01
Testosterone	< 0.001	< 0.001	0.77	.38
Monthly income	< − 0.01	0.02	0.16	.68
Education	0.01	0.01	1.29	.25
Relationship status			0.03	.86
yes/no	− 0.01	0.08		

For each variable, the estimate (*β*), standard error of the mean (SE), the *F* and the *p* values associated from the Fisher test of the comparison between the full model and the model without the factor are given. For the categorical variables “SexOr” and “Relationship status,” the estimates are given for one category compared to the reference category (SexOr: Homosexual men; Relationship status: No). *R*^2^ is the variance explained by the model. Sample size: *N*_Heterosexual men_ = 48; *N*_Homosexual men_ = 58, *N*_women_ = 54

**Table 5:** Linear model examining the influence of sexual orientation and sex on HNR

*R*^2^ = 58.8%	*Β*	SE	*F*	*P*
Intercept	10.79	0.22		
SexOr			55.64	< .001
Heterosexual men/homosexual men	− 0.77	0.26		
Heterosexual women/homosexual men	2.83	0.34		
Age	− 0.04	0.02	3.44	.06
Testosterone	< 0.001	< 0.01	0.02	.88
Monthly income	0.02	0.05	0.22	.63
Education	− 0.04	0.03	1.52	.22
Relationship status			0.18	.67
Yes/no	0.09	0.21		

For each variable, the estimate (*β*), standard error of the mean (SE), the *F* and the *p* values associated from the Fisher test of the comparison between the full model and the model without the factor are given. For the categorical variables “SexOr” and “Relationship status,” the estimates are given for one category compared to the reference category (SexOr: Homosexual men; Relationship status: No). *R*^2^ is the variance explained by the model. Sample size: *N*_Heterosexual men_ = 48; *N*_Homosexual men_ = 58, *N*_women_ = 54

**Fig. 1:**
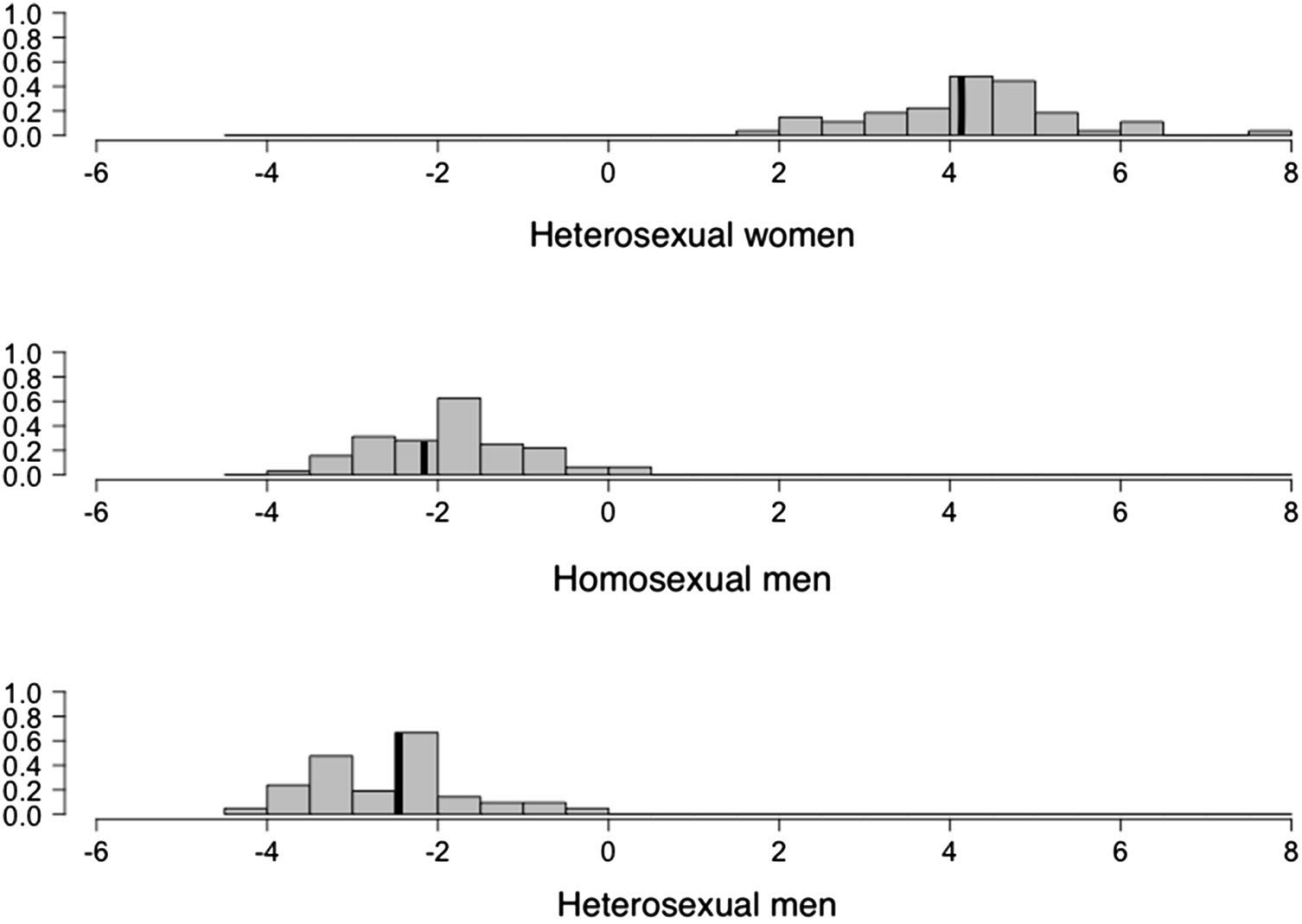
Distributions’ histograms of heterosexual women and homosexual and heterosexual men computed from the coordinates of the first linear discriminant function. The *Y*-axis represents the frequency and the *X*-axis the coordinates. Vertical solid lines represent the mean of each group (*N*_Heterosexual men_ = 48; *N*_Homosexual men_ = 58, *N*_women_ = 54)

The LDA separated the three groups using two discriminant functions: The first achieved 97.91% of the separation between the groups, whereas the second achieved only 2.09%. Coordinates were then computed from these functions. Since the second function could not accurately discriminate between the two sexes using their coordinates (*F*(1, 158) = 0.08, *p* = .77), the overall acoustic difference between the three groups was assessed using the coordinates of the first function. Post hoc comparisons revealed significant differences between all three groups: heterosexual and homosexual men (mean difference = − 0.71, *t*(157) = − 3.69, *p* < .001), heterosexual men and heterosexual women (mean difference = − 6.76, *t*(157) = − 33.78, *p* < .001), and homosexual men and heterosexual women (mean difference = − 6.05, *t*(157) = − 33.65, *p* < .001). Homosexual men showed a total of 10.65% differences in overall speech acoustic features compared to heterosexual men, slightly but significantly shifting toward those of heterosexual women (Fig. 1).

## Discussion

This study offers an interesting take on the interaction between sexual orientation and acoustic features of speech in a French speaker sample. First, our analysis of different acoustic features revealed well-known patterns of sexual dimorphism in human voices (i.e., F0, F0-SD, jitter, and HNR). Secondly, our findings showed that French homosexual men displayed a more modulated and less breathy voice than French heterosexual men, thus supporting and extending previous studies conducted mostly with English speakers. Our results for the LDA showed that French homosexual men attested a slight but significant vocal feminization when considering speech acoustic features altogether (up to 10.65%), which support the feminization hypothesis. (It is important to note, however, that no overlap was observed between heterosexual and homosexual men vs. heterosexual women.) Lastly, testosterone levels did not mediate the association between vocal patterns and sexual orientation.

Consistent with previous findings in English-speaking populations, no significant differences were observed in mean F0 between French-speaking heterosexual and homosexual men (Gaudio, 1994; Lerman & Damsté, 1969; Munson et al., 2006b; Rendall et al., 2008; Rogers et al., 2001; Smyth et al., 2003). The results did show a difference between homosexual and heterosexual men in intonation, the former displaying higher pitch variations than the latter. The relationship between pitch variations and sexual orientation was previously found in one Dutch (Baeck et al., 2011) and one American-English population (Gaudio, 1994), suggesting that feminized pitch variations might be characteristic of male homosexual speech across languages (but see Levon, 2006). In our study, the average difference in pitch variations reached ~ 4.11 Hz, which is largely above the just noticeable difference for pitch (Pisanski & Rendall, 2011). Hence, our findings suggest that pitch variations could be one of the acoustic correlates of sexual orientation that is used by listeners when they correctly assessed sexual orientation through speech only (Gaudio, 1994; Linville, 1998; Smyth et al., 2003; Valentova & Havlíček, 2013). Further investigations are nevertheless needed to confirm if such a difference in pitch variations between homosexual and heterosexual men is enough to be used as a cue for assessing sexual orientation.

To our knowledge, this is the first study to report an association between men’s vocal breathiness and sexual orientation. Interestingly, vocal breathiness has been suggested to be an important component of vocal femininity in female voices (Van Borsel et al., 2009) and significant relationships to vocal attractiveness have been reported in both sexes (Xu et al., 2013). Although the difference in vocal breathiness between homosexual and heterosexual men is rather low (mean average difference reached ~ 0.80 dB), further research should test whether it is perceptible by listeners to assess male sexual orientation and whether homosexual men’s voices, which are richer in harmonics compared to those of heterosexuals, are perceived as more attractive among homosexual men.

In our study, T-levels did not influence any of the acoustic parameters investigated. The methods to measure T-level and the sample size used in this study were similar to those used in previous studies finding a significant negative link between T-levels and F0 (e.g., Dabbs & Mallinger, 1999; Evans et al., 2008). However, testosterone is a multiple-effect hormone under the influence of numerous biological and environmental factors and pathways. As such, it is generally difficult to correlate T-levels with other biological or behavioral traits, especially with a unique measurement as realized here. Nevertheless, our results might suggest that other underlying processes, different than basal T-level, are involved in vocal differences between homosexual and heterosexual men.

Although our study does not aim to provide an explanation for why vocal differences were found between homosexual and heterosexual men, several biological and social mechanisms can be invoked. For instance, exposure to prenatal testosterone has been suggested to be responsible for the differences between homosexual and heterosexual men on a large range of characteristics such as physiological and behavioral traits including speech characteristics (Balthazart, 2017; Ehrhardt & Meyer-Bahlburg, 1981). Several studies have thus tested whether the 2D:4D ratio (relative length of the second and fourth digits), a proxy of testosterone prenatal exposure differs between homosexual and heterosexual men (Balthazart, 2017; Ehrhardt & Meyer-Bahlburg, 1981). However, there is currently no consensus regarding whether the 2D:4D ratio differs between heterosexual and homosexual men as studies have yielded mixed results (Breedlove, 2017; Grimbos, Dawood, Burriss, Zucker, & Puts, 2010; Rahman & Wilson, 2003; Robinson, 2000; Skorska & Bogaert, 2017; Williams et al., 2000). Regarding social mechanisms, a social imitation of women’s speech peculiarities by homosexual men could also explain the differences observed between homosexual and heterosexual men’s speech characteristics (at least for F0-SD and HNR). The use of more feminine acoustic characteristics by homosexual men could reflect a selective adoption model of opposite-sex speech patterns or a selective use of acoustic features for signaling in-group identity (Pierrehumbert et al., 2004), an ability called “gaydar” (i.e., the detection of homosexuality based on a set of specific cues). Interestingly, a recent study suggests that the acquisition of a distinctive speech style may happen before puberty, as boys aged from 5 to 13 with gender identity disorder (a diagnosis made when a child shows distress or discomfort due to a mismatch between his/her gender identity and his/her biological sex) display distinctive speech features (higher F0 and F2 as well as a misarticulation of/s/) from boys without it (Munson, Crocker, Pierrehumbert, Owen-Anderson, & Zucker, 2015). Because some homosexual men display a greater degree of gender nonconforming behavior (GNC) than others during childhood (Bailey & Zucker, 1995), one could thus hypothesize that the former would be more likely to have a more feminine speech in adulthood than the latter. Further work should investigate the relative importance of the mechanisms underlying homosexual men’s speech.

To conclude, although our study did not aim to test specific hypotheses against a formal theoretical framework to understand the differences between homosexual and heterosexual men’s speech, it provides some new descriptive findings. By examining for the first time native French speakers and some understudied acoustic features (i.e., namely, jitter and HNR), our results indicated that some vocal traits differed between heterosexual and homosexual men (i.e., variations of pitch and vocal breathiness) with values shifted toward heterosexual women’s vocal characteristics. Combined with the literature conducted in other languages, our findings bring new support for the feminization hypothesis (at least for some acoustic features) and suggest that the feminization of some acoustic features could be shared across languages. Further studies are needed to test whether intonation and vocal breathiness are perceptually salient to distinguish homosexual and heterosexual men, and whether overall differences are due to biological and/or sociolinguistic reasons.

## Data Availability

Data can be freely accessible at 10.6084/m9.figshare.11826636.v1
